# Does the Efficacy of Behavior Management Techniques Differ Between Children From Single-Child and Multi-Child Families?: A Quasi-Experimental Study

**DOI:** 10.3389/fpubh.2022.840483

**Published:** 2022-03-07

**Authors:** Lina Dai, Tingting Wu, Yun Hu, Shunyi Li, Weiwei Liu

**Affiliations:** ^1^Stomatological Hospital of Chongqing Medical University, Chongqing, China; ^2^Chongqing Key Laboratory of Oral Diseases and Biomedical Sciences, Chongqing, China; ^3^Chongqing Municipal Key Laboratory of Oral Biomedical Engineering of Higher Education, Chongqing, China; ^4^Chongqing Collaborative Innovation Center for Functional Food, Chongqing University of Education, Chongqing, China; ^5^Department of Food and Nutrition, College of Medical and Life Sciences, Silla University, Busan, South Korea; ^6^Reasearch Center for Public Health Security, College of Public Health and Management, Chongqing Medical University, Chongqing, China

**Keywords:** behavior management techniques, children's dental anxiety, compliance, single-child, multi-child

## Abstract

**Aim:**

Behavior management techniques (BMTs) efficiently deliver dental treatment to children with dental anxiety. The objective of this quasi-experimental study was to examine whether the efficacy of BMTs applied for the improvement of compliance in pediatric patients differs between children 3–10-year-olds from single-child and multi-child families.

**Materials and Methods:**

In this quasi-experimental, 197 caregiver-child couples were divided into two groups: single-child group (116 couples) and multi-child group (81 couples). Children's pre- and post-treatment anxiety levels were measured by facial mood scale (FMS) and Frankl Behavior Rating Scale (FBRS), respectively.

Caregivers' dental anxiety was measured by the Chinese version of the Modified Dental Anxiety Scale (MDAS), which was included in the self-designed questionnaire. Data were analyzed by using the Mann-Whitney *U*-test, chi-square tests, and binary multivariate regression analysis.

**Results:**

There was no statistically significant difference in the demographic characteristics of the children between the two groups. BMTs were found to be capable of reducing children's dental anxiety (CDA): the compliance rate was 45.69–88.79% in the single-child group and 44.44–85.79% in the multi-child group pre- and post-BMTs, but there was no significant difference in the change of compliance between the two groups (*p* > 0.05). In the subgroup analysis, parenting style (odds ratio [OR] = 0.054, *p* < 0.05) and father's education (OR = 8.19, *p* < 0.05) affected the varies of children's compliance in the single-child group. In contrast, in the multi-child group, gender (OR = 8.004, *p* < 0.05) and mother's occupation (OR = 0.017, *p* < 0.05) were associated with these changes in compliance.

**Conclusions:**

In this study, BMTs were proved to be beneficial in improving compliance in 3- to 10-year-olds children in dental treatment. Though there was no significant difference in the change of compliance between children from single-child and multi-child families, different associated factors may affect the two groups. Therefore, the related family factors should be taken into account when professionals manage each child's behavior in dental practice.

## Introduction

Children's dental anxiety (CDA) refers to a feeling or anticipation that something will happen, combined with a sense of losing control to dentistry, which is one of the major challenges in pediatric dentistry (1). Due to the different target populations and designs, the prevalence of CDA among children was reported differently: ranging from 6.3 (2) to 93.8% (3). The vicious cycle theory is proposed that dental anxiety plays an important role in the dental avoidance pattern, which in turn, leads to untreated diseases, causing the deterioration of dental anxiety (4). Childhood dental anxiety was proved as one of the predictors associated with oral health-related quality of life (OHRQOL) *via* path analysis (5). Several interacting complicated etiologies may contribute to the acquisition of CDA, such as age, culture, environment, psychology, cognition, and family factors (6–12). Moreover, the term “dental anxiety” is often used to include all types of dental fear and phobias (13). How to prevent or intercept CDA remains a great challenge for dental professionals.

A clinical phenomenon attracted us: though some pediatric patients exhibited high scores of dental anxiety, they still had the potential to complete the dental treatment, however, other children seemed not to be able to overcome their dental anxiety, which caused behavior management problems (BMPs). It is indicated that some children can have hidden dental anxiety to overcome their resistance behaviors and CDA is manageable (14). Behavior management techniques (BMTs) are applied to alleviate fear and anxiety, especially in children, which are considered integral components in pediatric dentistry (15). Previous studies have demonstrated that BMTs were effective to alleviate dental anxiety, such as music distraction (MD) (16), audiovisual distraction (AVD) (17), Tell show do (TSD)(18), “little lovely dentist” (19), and so on. It was suggested that combined BMTs for reducing anxiety in children have a better result rather than a single procedure alone (20).

One-child Policy (OCP) was adopted by China's government in 1979, which significantly changed people's risk preferences, and social preferences reshaped Chinese society (21). Following the end of OCP in China in 2016, the possibility of adding another child into the family may have profound implications on the family system (22). As the triple relationship among dental professionals, children, and parent(s), the efficacy of BMTs is also closely associated with these factors, such as the presence of siblings. It has been demonstrated that children's birth order partially determines one's personality and behavior in medical situations (23). In addition, the child is more likely to have CDA, if his sibling was reported CDA (6). While Peretz's study showed that no association was found between children's dental fear and the number of children in their families (24). The reports on whether there is an association between the efficacy of BMTs and the presence of siblings were still few.

This study was the first to focus on the efficacy of BMTs in children from single-child and multi-child families in Chongqing and explored the possible influencing factors in the two styles of families. The aim of our study was to assist clinicians to provide better oral health services for their pediatric patients.

## Materials and Methods

### Sampling

Concerning the effect evaluation of behavior management in children's oral diagnosis and treatment in Chongqing, the effectiveness was 93% (25). The sample size was calculated by using G^*^Power version 3.1.9.2. Targeting a statistical power of 0.95 and a significant level of 0.05 and estimating 93%, 10% of non-response, the total sample taken was 110. Inclusion criteria included pediatric patients of 3–10 years old with a dental history of no more than three appointments and caregivers can use cell phones. Exclusion criteria were history of chronic disease and mental disorder. A total of 197 pairs of children and their caregivers recruited in this study were divided into two groups: 116 couples from single-child families and 81 couples from multi-child families.

### Methodology

The quasi-experimental trial was done at the Department of Pediatric Dentistry, Stomatological Hospital of Chongqing Medical University between May and November 2021. Before recruiting participants for this study, permission was received from caregivers' authorities, and the study was explained in detail. If the caregivers showed the entirely voluntary to participate in this study, the child would be selected. All caregivers were asked to give their written informed consent. The ethics committee approval of the Stomatological Hospital of Chongqing Medical University was obtained (CQHS-IRB-2021-38).

**Figure 1:**
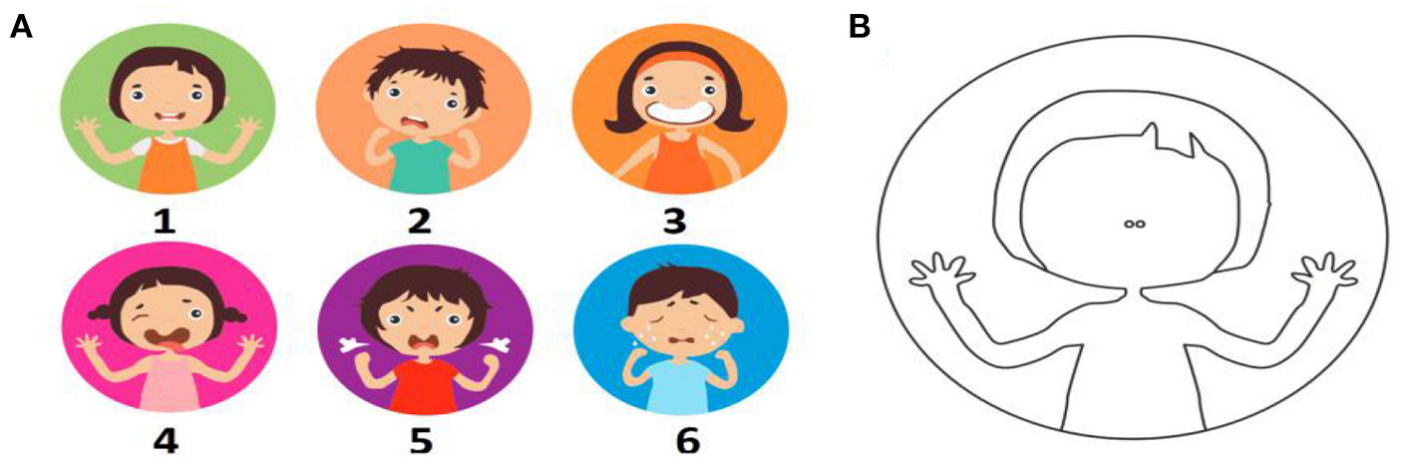
**(A)** Face mood scales used to assess the initial anxiety level of children pre-treatment**. (B)** A blank face was used to express children's own emotional state by drawing missing elements: eyes, nose. 1-calm; 2-uncertain; 3-reserved, closed and uncooperative; 4-avoiding; 5-loud; and 6-crying (26).

The pre- and post-treatment designs were used in the study. Before the treatment, all children were asked to complete the drawing by adding the facial elements, eyes, nose, and lips, with the help of a nurse, as shown in Figure 1B. The professionals then categorized the children's drawings to the corresponding faces 1–6 of facial mood scale (FMS), as shown in Figure 1A, mostly by matching eye and lip. The step may take about 5–10 min. The children were classified as “cooperative group” (1. calm; 2. uncertain) and “uncooperative group” (3. reserved; 4. avoiding; 5. loud; and 6. crying) according to the direction in FMS (26).

At the same time, the caregivers (parents in most cases) were provided a dental questionnaire related to demographic information and their dental anxiety *via* completion of the Chinese version of the Modified Dental Anxiety Scale (MDAS). The gender, age, caregivers, dental history, dental diagnosis, parenting styles, parent(s)'occupations, education, annual household income, and so on were recorded.

During dental treatment, the caregiver was separated from the child. Since protective stabilization was prohibited in the Department of Pediatric Dentistry and general anesthesia was not preferred by parents, MD (16), AVD (17), TSD (18), and combined other BMTs were adopted.

After the treatment, the dentist rated pediatric patients' behavior during the treatment according to Frankl Behavior Rating Scale (FBRS). To ensure sample “blindness”, the rater was unaware of the children's pre-treatment dental anxiety (TDA) and compliance. The children were classified as “cooperative group” (Rating 3. sometimes nervous but coordinate the treatment; Rating 4. respond well to treatment) and “uncooperative group” (Rating 1. definitely negative; Rating 2. avoiding) according to the direction in FBRS (14).

## Measurements

### Face Mood Scale

The Chinese face version of the modified child dental anxiety scale internal consistency coefficient was 0.814, the reliability was 0.907 (27), but there are five items in this version requiring children's verbal expression. Due to the limited verbal ability of children, a simple method was required to access the initial anxiety level of children in this study. Children's drawings were identified to evaluate dental anxiety, which was correlated with FBRS (28). Facial Action Coding System (FACS) was developed by Eckman and Friesen (29), it was based on the idea that each emotion can be associated with different facial muscle patterns and that by analyzing facial regions where these muscles are activated. Compared to other analysis methods, the main advantage of FACS is that it can determine hidden emotions, 87.2% accuracy for depression, 77.9% for anxiety, and 90.2% for stress (30). Children and their caregivers prefer the faces scale over other evaluation methods (31, 32). The method was used by Polish researchers to evaluate the children's anxiety level during the Coronavirus Disease-19 (COVID-19) pandemic in 2020 (26). All above provides the support for FMS used in this study.

### Frankl Behavior Rating Scale

Considering the children and caregivers' boredom emotion caused by answering the questionnaire one more time, we did not use the Chinese version of children's fear survey schedule-dental subscale (CFSS-DS) to access the children's compliance post-treatment. FBRS was used to examine the reliability and validity of the Chinese version of the CFSS-DS (33). Therefore, an experienced dentist was asked to classify children's behavior with FBRS instead of CFSS-DS after treatment. FBRS was widely used to access the behavior of children (34–36) and the effectiveness of BMTs in children's dental treatment (37, 38). The compliance scores were measured by using FBRS after treatment in this study. The classifications of FBRS are as followed:

Rating 1. Definitely negative: refusal of treatment, crying;

Rating 2. Negative: reluctant to accept treatment, uncooperative, some evidence of negative attitude but not pronounced;

Rating 3. Positive: acceptance of treatment, at times cautious, willingness to comply with the dentist, at times with reservation, but patient follows the dentist's directions cooperatively;

Rating 4. Definitely positive: good rapport with the dentist, interested in the dental procedures, laughing, and enjoying.

### Questionnaire

One month before the formal survey, we conducted a pre-survey collected 30 samples and revised the questionnaire based on these results. All participating parents signed the informed consent forms before the formal survey. Reliability and validity tests were used. The reliability of Cronbach's alpha coefficient of the questionnaire was 0.83. The Kaiser-Meyer-Olkin (KMO) validity statistical test (KMO = 0.916) and Bartlett sphericity test (*p* < 0.0001) were used. The questionnaire for this survey was prepared through consultations with epidemiologists and dentists regarding a large number of documents and consisted of two parts:

(1) Socio-demographic characteristics, i: information about children, such as age, gender, dental diagnosis, and dental visits; ii: information about main caregiver(s), dental fear level of caregiver, occupation and education of parents, parenting style, and annual household income;

(2) Chinese version of MDAS

The Chinese MDAS consisted of two factors: anticipatory dental anxiety (ADA) and TDA. Internal consistency coefficients (tau non-equivalent) were 0.74 and 0.86, respectively (39).

### Statistical Analysis

All these analyses were performed using Stata version 15.1 software (Stata, College Station, TX, USA). The dental anxiety score of children was measured on the ordinal scale, and the age of children did not meet the assumption of normal distribution (Shapiro-Wilk test; *p* < 0.05). Parametric and non-parametric tests were used for comparing means/medians, whereas chi-square tests were used for comparing proportions. Participants' socio-demographic profile and family-related factors were described. The difference in dental anxiety scores of children in single child and multi-children families was assessed with the Mann-Whitney U test. Chi-square tests were used for comparing proportions of dental anxiety behavior of cooperative and uncooperative groups. Binary multivariate regression analysis was used to rate the effectiveness for improving compliance and factors influencing the effectiveness of BMTs on single-child and multi-child families. The following three models were used. Model 1 adjusted for gender and age. Model 2 further adjusted for dental visits, dental disease, caregiver, and annual household income level. Model 3 further adjusted for parenting style, education level of father, occupation of father, the education level of mother, and the occupation of mother. The results are reported for the odds ratio (OR) and 95% CI.

## Results

Table 1 shows the characteristics of the caregiver-child couples in this study. In total, 58.88% of children were from single-child families, while 41.12% were from multi-child families. The gender distribution of children was relatively balanced, with 51.27% boys and 48.73% girls. More than half of pediatric patients in the research were suffered from dental caries. Approximately two-thirds of the caregivers in this survey were parents. More than 80% of the caregivers were reported to provide a balanced parenting style to their children and nearly 50% of the caregivers were reported to have dental fear. Over half of the families had an annual household income of more than >¥150,000. No statistically significant difference in the demographic characteristics was found between the two groups (*p* > 0.05).

**Table 1:** Characteristics of participants at baseline.

**Variables**		**Family**		**Total**	***P***
		**Multi-child family (*n* = 81)**	**Single-child family (*n* = 116)**	***N* = 197**	
Ag**e**	3–6years old	22 (27.16%)	42 (36.21%)	64 (32.49%)	0.23
	7–8 years old	38 (46.91%)	54 (46.55%)	92 (46.70%)	
	9–12 years old	21 (25.93%)	20 (17.24%)	41 (20.81%)	
Gender	Boy	40 (49.38%)	61 (52.59%)	101 (51.27%)	0.66
	Girl	41 (50.62%)	55 (47.41%)	96 (48.73%)	
Caregiver	Parents	63 (77.78%)	79 (68.10%)	142 (72.08%)	0.14
	Grandparents	18 (22.22%)	37 (31.90%)	55 (27.92%)	
Dental diagnosis	Dental caries	41 (50.62%)	66 (56.90%)	107 (54.31%)	0.68
	Endodontic treatment	11 (13.58%)	10 (8.62%)	21 (10.66%)	
	Tooth extraction	13 (16.05%)	17 (14.66%)	30 (15.23%)	
	Orthodontics and health observation	16 (19.75%)	23 (19.83%)	39 (19.80%)	
Dental visits	First time	21 (25.93%)	43 (37.07%)	64 (32.49%)	0.12
	Second time	20 (24.69%)	32 (27.59%)	52 (26.40%)	
	Third time	40 (49.38%)	41 (35.34%)	81 (41.12%)	
Occupation of father	Managerial or professional	12 (14.81%)	16 (13.79%)	28 (14.21%)	0.53
	Labor	40 (49.38%)	66 (56.90%)	106 (53.81%)	
	Clerical	16 (19.75%)	23 (19.83%)	39 (19.80%)	
	Freelance work	13 (16.05%)	11 (9.48%)	24 (12.18%)	
Occupation of mother	Managerial or professional	18 (22.22%)	19 (16.38%)	37 (18.78%)	0.69
	Labor	36 (44.44%)	57 (49.14%)	93 (47.21%)	
	Clerical	4 (4.94%)	4 (3.45%)	8 (4.06%)	
	Freelance work or housewife	23 (28.40%)	36 (31.03%)	59 (29.95%)	
Education of father	Bachelor degree below	27 (33.33%)	44 (37.93%)	71 (36.04%)	0.40
	Bachelor degree	37 (45.68%)	56 (48.28%)	93 (47.21%)	
	Master degree and above	17 (20.99%)	16 (13.79%)	33 (16.75%)	
Education of mother	Bachelor degree below	33 (40.74%)	51 (43.97%)	84 (42.64%)	0.89
	Bachelor degree	34 (41.98%)	47 (40.52%)	81 (41.12%)	
	Master degree and above	14 (17.28%)	18 (15.52%)	32 (16.24%)	
Annual	< ¥80,000	17 (20.99%)	16 (13.79%)	33 (16.75%)	0.38
household income	¥80,000–150,000	21 (25.93%)	36 (31.03%)	57 (28.93%)	
	>¥150,000	43 (53.09%)	64 (55.17%)	107 (54.31%)	
Parenting style	Authoritative	68 (83.95%)	100 (86.21%)	168 (85.28%)	0.091
	Permissive	10 (12.35%)	6 (5.17%)	16 (8.12%)	
	Authoritarian	3 (3.70%)	10 (8.62%)	13 (6.60%)	
Dental fear	None	47 (58.02%)	65 (56.03%)	112 (56.85%)	0.22
level of caregivers	Mild to moderate	25 (30.86%)	28 (24.14%)	53 (26.90%)	
	Sever	9 (11.11%)	23 (19.83%)	32 (16.24%)	

*Data are presented as mean ± SD for continuous measures, and n (%) for categorical measures*.

**Figure 2:**
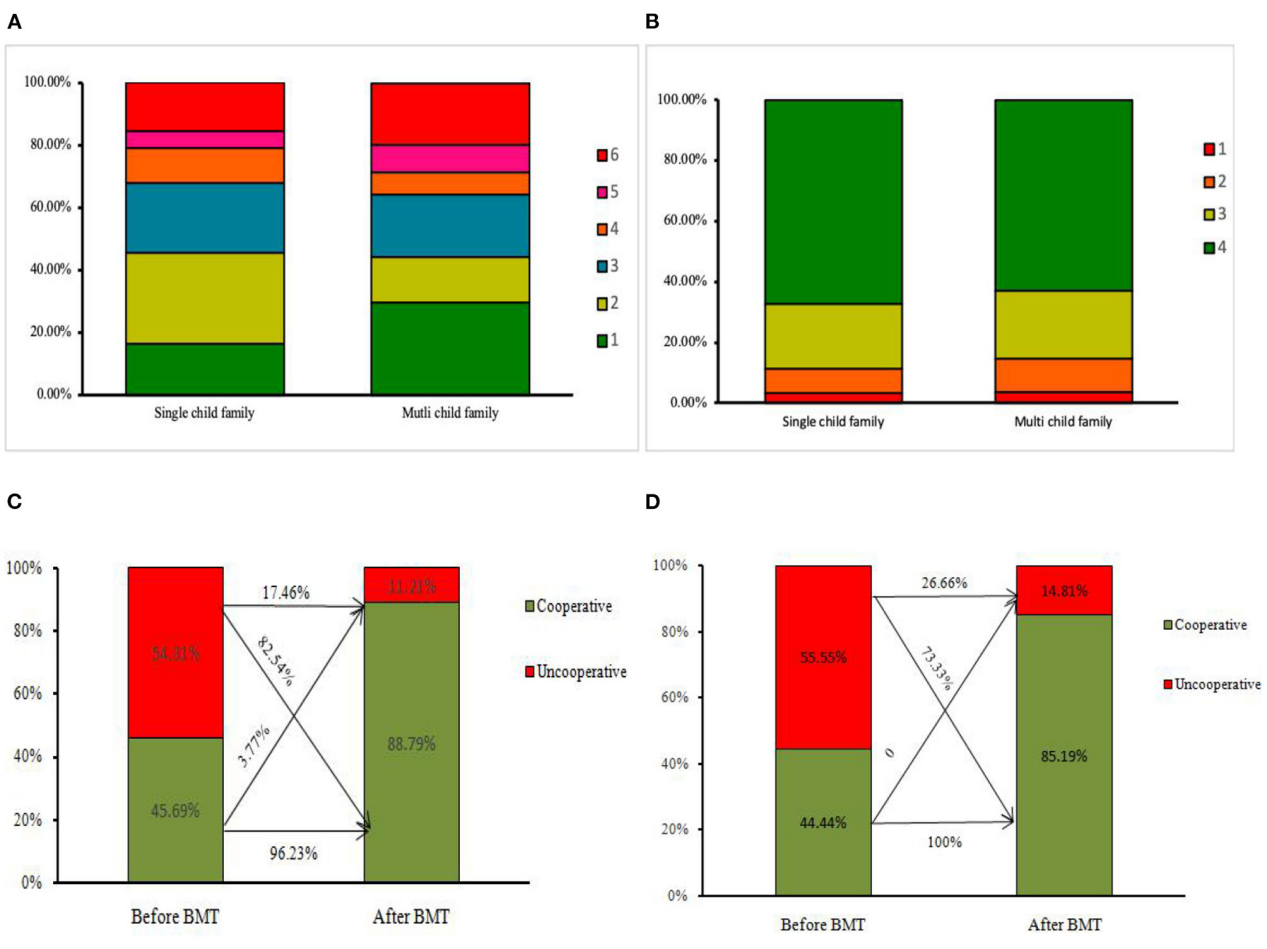
**(A)** The scores of facial mood scale (FMS) pre-treatment in two groups. **(B)** The scores of Frankl Behavior Rating Scale (FBRS) post-treatment in two groups. **(C)** The varies of the proportion of cooperative children in the single-child group. **(D)** The varies of the proportion of cooperative children in the multi-child group.

As demonstrated, before BMTs, the FMS score of children from single-child family did not reveal a different anxiety level as compared to children from multi-child families [median (inter-quartile range, IQR): 3 (2–4) vs. 3 (1–5)], (*p* = 0.8116, Mann-Whitney *U*-Test). However, the shares of children scoring the highest anxiety score of 6 in the single-child family group and the multi-child family group were 15.04 and 19.75%, respectively. Moreover, the lowest dental anxiety scores of 1 in two types of families were 16.38 and 29.60%. The summary of scores per- and post-treatments in two groups is presented in Table 2 and Figures 2A,B.

**Table 2:** The score of facial mood scale (FMS) pre-treatment and the score of Frankl Behavior Rating Scale (FBRS) post-treatment.

**FMS/FBRS score**	**Total**	**Single-child family (*n* = 116)**	**Multi-child family (*n* = 81)**	***p*-value**
Median (IQR) FMS scores	3 (2–4)	3 (2–4)	3 (1–5)	0.812
1	21.83%	16.38%	29.60%	
2	23.35%	29.31%	14.80%	
3	21.32%	22.41%	19.70%	
4	9.64%	11.21%	7.40%	
5	6.60%	5.17%	8.60%	
6	17.26%	15.52%	19.70%	
Median (IQR)FBRS Score	4 (3, 4)	4 (3, 4)	4 (3, 4)	0.489
1	3.55%	3.45%	3.70%	
2	9.14%	7.76%	11.11%	
3	21.83 %	21.55 %	22.22%	
4	65.48%	67.24%	62.96%	

*n (%) for categorical measures*.

The proportion of cooperative children was increased significantly in both the single-child group and multi-child group post-treatments (*p* = 0.001 vs. *p* = 0.002). The proportion of cooperative children in the single-child group and multi-child group was 45.69 and 44.44%, the difference was not significant (*p* = 0.863). 82.5% of uncooperative children were guided to cooperative after BMTs in the single-child family group, while the proportion was 73.33% in multi-child family group, which was shown in Table 3 and Figures 2C,D.

**Table 3:** The proportion of cooperative children in single-child group and multi-child group.

	**Before BMTs**		**After BMTs**		**Δ**	***p* ^#^**
	**Cooperative**	**Uncooperative**	**Cooperative**	**Uncooperative**		
Single- child group	53 (45.69%)	63 (54.31%)	103 (88.79%)	13 (11.21%)	82.5%	0.001
Multi-child group	36 (44.44%)	45 (55.56%)	69 (85.19%)	12 (14.81%)	73.33%	0.020
*P* ^##^	0.863		0.454			

*p <0.05. Δ: The varies in the proportion of compliance in the two groups.

# *Comparison between before and after behavior management techniques (BMTs) in the same family group*.

## *Comparison between single-child group and multi-child group*.

The OR for the effectiveness of BMTs in improving compliance in single-child and multi-child groups is shown in Table 4. We found that the OR of BMTs in improving compliance of children from the single-child group was higher than that of children from the multi-child group, while the difference was not statistically significant in all three models (*p* > 0.05).

**Table 4:** Odds ratio (OR; 95% CI) the for the effectiveness of behavior management techniques (BMTs) in improving compliance in single-child group and multi-child group.

**Models**	**Types of family**	
	**Multi-child family**	**Single-child family**
Model 1	1	1.179 (0.652, 2.134)
Model 2	1	1.374 (0.735, 2.572)
Model 3	1	1.807 (0.894, 3.651)

*p <0.05, model 1 adjusted for gender and age. Model 2 adjusted for gender, age, dental visits, dental disease, the dental anxiety level of caregiver, and annual household income level. Model 3 adjusted for g gender, age, dental visits, dental diagnosis, the dental anxiety level of caregiver, annual household income level, parenting style, education of father/mother, and occupation of father/mother.

Table 5 shows the factors that influence the effectiveness of BMTs in the single-child group and multi-child group. In the single-child group, children whose caregivers provided a balanced parenting style were reported to more likely to be cooperative after BMTs (OR = 0.054, 95% CI: 0.004, 0.771); children whose father with a bachelor degree had 8.195 times higher OR to be cooperative after BMTs than those whose fathers with a degree lower than a bachelor.

**Table 5:** Factors that influence the effectiveness of behavior management techniques (BMTs) in improving compliance in single-child and multi-child group.

**Variables**	**Sub-group**	**Types of family [odds ratio (95%CI)]**	
		**Single-child family**	**Multi-child family**
Gender	Boy	1	1
	Girl	0.782 (0.278, 2.2)	8.004 (1.483, 43.196)^*^
Age	3–6years old	1	1
	7–8years old	0.939 (0.284, 3.107)	0.446 (0.079, 2.509)
	9–12 years old	1.286 (0.232, 7.143)	0.87 (0.106, 7.151)
Dental visits	First time	1	1
	Second time	1.658 (0.453, 6.067)	0.239 (0.026, 2.186)
	Third time	1.104 (0.292, 4.177)	0.462 (0.079, 2.696)
Dental diagnosis	Dental caries	1	1
	Endodontic treatment	1.286 (0.235, 7.038)	1.749 (0.0165, 18.517)
	Tooth extraction	0.458 (0.074, 2.839)	1.675 (0.261, 10.75)
	Orthodontics and health observation	1.235 (0.331, 4.606)	0.166 (0.021, 1.315)
Caregivers	Parents	1	1
	Grandparents	0.296 (0.087, 1.009)	1.605 (0.158, 16.319)
Annual household income	< ¥80,000	1	1
	¥80,000–150,000	2.064 (0.342, 12.469)	0.288 (0.034, 2.456)
	>¥150,000	2.477 (0.397, 15.459)	0.658 (0.088, 4.909)
Parenting style	Authoritative	1	1
	Permissive	4.448 (0.721, 8.986)	1.78 (0.192, 16.494)
	Authoritarian	0.054 (0.004, 0.771)^*^	0.584 (0.017, 20.03)
The dental anxiety level of caregiver	None	1	1
	Mild to moderate	0.707 (0.22, 2.278)	1.463 (0.234, 9.147)
	Sever	1.85 (0.442, 7.744)	0.774 (0.073, 8.165)
Occupation of father	Managerial or professional	1	1
	Labor	2.076 (0.407, 10.595)	0.909 (0.064, 12.921)
	Clerical	5.939 (0.551, 0.64)	3.245 (0.139, 75.875)
	Freelance work	8.647 (0.969, 58.993)	5.364 (0.18, 159.446)
Occupation of mother	Managerial or professional	1	1
	Labor	0.54 (0.124, 2.351)	0.187 (0.017, 2.017)
	Clerical	6.755 (0.24, 17.966)	0.135 (0.005, 3.999)
	Freelance work or housewife	0.32 (0.041, 2.501)	0.017 (0.001, 0.428)^*^
Education of father	Bachelor degree below	1	1
	Bachelor degree	8.195 (1.798, 37.346)^*^	2.09 (0.284, 15.359)
	Master degree and above	6.282 (0.726, 54.385)	0.355 (0.034, 3.659)
Education of mother	Bachelor degree below	1	1
	Bachelor degree	1.943 (0.477, 7.912)	3.789 (0.658, 21.82)
	Master degree and above	0.678 (0.105, 4.36)	0.334 (0.026, 4.333)

* *p <0.05, the full model is adjusted for gender, age, dental visits, dental diagnosis, the dental anxiety level of caregiver, annual household income level, parenting style, education of father/mother, occupation of father/mother*.

Interestingly, unlike in the single-child group, we found that children's gender influenced the effectiveness of BMTs: girls were more likely to be cooperative (OR = 8.004, 95% CI: 1.482, 13.196) than boys in the multi-child group. Children whose mother's occupation is freelance work or housewife were less likely to be cooperative after BMTs than those whose mothers are managerial or professionals (OR = 0.017, 95% CI: 0.001, 0.428).

## Discussion

The two-child policy was implemented in China in 2016, and policy was shifted into three children in 2021, the new policy has contributed to socioeconomic changes and family structure varies (40). The surveys of fertility preference undertaken over the past two decades in China showed that nearly two-thirds of women in large cities stated a preference for only one child (40), considering the high cost of parenting and the impact on parenting styles and mothers' careers (41). The presence of siblings has been a significant determinant for CDA (6). But what was the association about the effectiveness of BMTs with the presence of siblings? Our study aimed to explore the effectiveness of BMTs in reducing dental anxiety in children from single-child and multi-child families and to assess influencing factors in the two types of families.

Different methods and different target populations might cause a difference in the prevalence of CDA. In this study, we found that 54.12% of children were suffering from dental anxiety by FMS score, which is consistent with our previous study in Chongqing (42), while significantly higher than the prevalence of CDA in 2017 using CFSS (43, 44) and higher than the prevalence of CDA of Poland during COVID-19 period in 2020 (26). This might suggest a high level of dental anxiety among children in the Chongqing area, requiring appropriate methods to alleviate it.

This study demonstrated that BMT is effective in improving compliance of children, which is consistent with previous studies (15, 45). CDA is not only associated with pain or invasive procedures but also correlated with confronting unfamiliar persons or environments (46). MD and AV methods of BMTs provide a comfortable environment for children when seeing a dentist, which was applied to distract children's attention and reduce children's anxiety (47, 48). TSD was used to manage children's behavior by visual-body language and other methods to help them understand the treatment process (49, 50). The results indicated that there was no significant difference in the effectiveness of BMTs between the single-child group and the multi-child group, suggesting the presence of siblings might not contribute to the efficiency of BMTs. To solve practical problems based on the findings of clinical work, we still wanted to explore whether the factors influencing the efficacy of BMTs in these two types of families were different. Thus, the subgroup analysis had some interesting findings.

Though a report in Jeddah, Saudi Arabia showed that the gender difference was not found after BMTs (51), the finding of this study showed the effectiveness of the BMTs on girls is likely greater than boys in the multi-child group. This might be related to earlier physical and mental development, girls may have better cognitive development (52). It was believed that girls are more adult-oriented and affected by parental factors and less by their siblings (6). Moreover, this may lead to more affected by dentists managing their behavior. The results of this study suggest clinicians or parents should pay more attention to boys in multi-child families in the future, which may improve the efficacy of BMTs.

Although some studies showed no significant difference was found in fear levels of children with their parents' education, occupation (44). In the present study, children whose mothers' occupations were freelance or housewives were less likely to change their behavior compared to children whose mothers' occupations were managerial or professional in the multi-child group. We speculate that this may be related to the fact that managerial or professional women are more economically independent and better at decision-making.

On the other hand, mothers who are housewives or freelancers may spend more time with their children, whose children may be less independent and easy to be shy to a stranger. This finding suggested that children from multi-child families whose mothers are freelance or housewives may need more attention and encouragement during dental treatment, meanwhile, it is necessary to strengthen the oral health education for their mothers.

A systematic review supported that there was a relationship between parenting style and children's dental anxiety and their behavior, although this relationship was limited to pre-school children with no dental experience or dental phobia (53). In the single-child group, children who received an authoritative parenting style were found more likely to change their behavior after BMTs than those who received an authoritarian parenting style. Authoritative parenting, that is, the combination of high warmth with limit-setting, has been linked to children with improved psycho-social maturity, positive behavior, more academic competence, less internalized distress, and less externalizing problems (44). In conclusion, parenting style should be taken into account as the content of dentist-parents consultation and considered as the selection of behavior guidance techniques, which might improve the effectiveness of BMTs.

Parents with higher education tended to have better oral health knowledge (54). As to children's oral problems, well-educated mothers will not let their children lack dental care; they will be more inclined to instill more relevant information to dispel their children's anxiety (55). In the present study, father's education was found to be significantly associated with the efficacy of BMTs in the single family group. This may be related to the recent shift in family structure in modern society, as women have begun to enter the workforce more often and men have correspondingly taken on more responsibility for family education, especially in one-child families (56). Studies point out that fathers take more interactive caresses, such as talking, speaking, and teaching, when staying with children (57). Furthermore, the importance of fathers' role on child dental fear has been studied to a less extent, the influence of fathers on dental fear and behavior management may be a topic worthy of continued exploration.

The present study is one of few reports to explore the effectiveness of BMTs in reducing dental anxiety and the associated factors that may influence the effectiveness in the single-child families and multi-child families. Some limitations existed in the study. Firstly, the quasi-experimental study could not investigate the causal relationship of the factors with the effectiveness of BMTs. Secondly, as a convenient sampling technique, the findings cannot be generalized and straightforward to different populations of China, limited to the target population being children who visited the dental clinic for treatment in Chongqing. Thirdly, the recall bias of the questionnaire was another limitation, even the parents were given enough time to recall and answer the questionnaires and the questions were designed to ask about a recent period. Additionally, because they may tend to give socially desirable answers, some caregivers' perception may not reflect the reality, further research studies are worth continuing.

## Conclusion

Behavior management techniques are effective in reducing dental anxiety, although, the effectiveness of BMTs might not be significantly different between children from single-child families and multi-child families. However, factors that influenced the effectiveness of BMTs were significantly different between the two types of families. Gender and maternal occupation had a significant effect on the change of compliance in the multi-child group, whereas the father's education and parenting style were found to be associated with the change of compliance in the single-child group. This study suggested that obtaining comprehensive family and socio-demographic factors from children or parents is as important as consulting dental history. This information may assist professionals to make appropriate plans and take effective BMTs for their pediatric patients, especially in the current society and era of China, the number of children will change due to the government's population policy. It is beneficial to pediatric patients if professionals master that children from different types of families may need different BMTs, to some extent, which will make it easier for dentists to guide children to control the CDA.

## Data Availability Statement

The original contributions presented in the study are included in the article/supplementary material, further inquiries can be directed to the corresponding author.

## Ethics Statement

The studies involving human participants were reviewed and approved by Stomatological Hospital of Chongqing Medical University was obtained (CQHS-IRB-2021-38). Written informed consent to participate in this study was provided by the participants' legal guardian/next of kin.

## Author Contributions

WL and LD: study design. LD: data collection. TW: statistical analysis. YH and SL: data interpretation. LD and TW: manuscript preparation. YH, SL, LD and TW: literature search. WL: funds collection. All authors contributed to the article and approved the submitted version.

## Funding

This study received funding from Humanities and Social Sciences Research project of Chongqing Municipal Commission of Education (21SKJD042).

## Conflict of Interest

The authors declare that the research was conducted in the absence of any commercial or financial relationships that could be construed as a potential conflict of interest.

## Publisher's Note

All claims expressed in this article are solely those of the authors and do not necessarily represent those of their affiliated organizations, or those of the publisher, the editors and the reviewers. Any product that may be evaluated in this article, or claim that may be made by its manufacturer, is not guaranteed or endorsed by the publisher.
